# Low-Molecular-Weight Heparin in Orthopedic Patients Taking Clopidogrel: A Focused Review

**DOI:** 10.7759/cureus.94333

**Published:** 2025-10-11

**Authors:** Ahmed Mohamed, Abed Ullah Khan, Usman Fuad, Alaa Elasad

**Affiliations:** 1 Trauma and Orthopaedics, Royal Cornwall Hospital, Truro, GBR; 2 Spinal Surgery and Neurosciences, Leeds Teaching Hospital, Leeds, GBR; 3 General Practice, Zagazig University, Zagazig, EGY

**Keywords:** antiplatelet therapy, arterial thrombosis, bleeding risk, clopidogrel, hip fracture, low-molecular-weight heparin, orthopedic surgery, perioperative anticoagulation, venous thromboembolism prophylaxis, venous thrombosis

## Abstract

Orthopedic surgeons frequently manage patients taking clopidogrel who require low-molecular-weight heparin (LMWH) for venous thromboprophylaxis. This review examines the differences between arterial and venous thrombosis and explains why both medications are often necessary despite the increased bleeding risk. We conducted a comprehensive literature search of PubMed, Cochrane Library, Embase, and Google Scholar from January 2000 to August 2025, screening 1,184 records and ultimately including 57 studies comprising eight randomized controlled trials, 32 cohort studies, and 17 systematic reviews. Quality assessment was performed using the Cochrane Risk of Bias tool and Newcastle-Ottawa Scale. Due to substantial heterogeneity in study populations, interventions, and outcome definitions, we conducted structured narrative synthesis rather than meta-analysis. We provide clear guidance on when to stop or continue clopidogrel therapy in elective versus trauma surgery and how to safely combine it with LMWH when needed. Key recommendations include that clopidogrel alone does not prevent venous thromboembolism, LMWH remains necessary in immobilized patients, elective surgery usually requires the temporary cessation of clopidogrel, and trauma surgery should not be delayed despite ongoing therapy.

## Introduction and background

Many orthopedic patients are prescribed clopidogrel for cardiovascular protection purposes. After orthopedic injuries or surgeries, when patients are prohibited from weight-bearing, surgeons face difficulties in dealing with patients who are on antiplatelet treatment. Should low-molecular-weight heparin (LMWH) be added for venous thromboembolism (VTE) prevention? Can clopidogrel alone provide adequate protection? Understanding the answer requires recognizing that arterial and venous clots form through different mechanisms [1].

Clopidogrel prevents arterial thrombosis by inhibiting platelet aggregation. It protects against heart attacks and strokes but does not prevent venous clot formation. LMWH prevents venous thrombosis by enhancing anticoagulation; however, it does not protect against arterial events. Orthopedic patients who become immobilized are at risk of venous clots known as deep vein thrombosis (DVT), which clopidogrel cannot prevent. This creates a dilemma in which both medications may be necessary despite an increased bleeding risk [2-4].

This review provides practical guidance for prescribing LMWH to patients taking clopidogrel. We explain why both medications target different types of clots, examine the bleeding risks of combination therapy, and provide recommendations for stopping or continuing clopidogrel in different surgical scenarios.

## Review

Methods

We conducted a focused literature review to provide practical guidance on the perioperative management of orthopedic patients receiving clopidogrel who require VTE prophylaxis. Our search covered multiple medical databases, including PubMed, Cochrane Library, Embase, and Google Scholar, for papers published from January 2000 to August 2025. We used search terms combining concepts related to clopidogrel and antiplatelet therapy, orthopedic and trauma surgery, LMWH and anticoagulation, and outcomes including bleeding risk and thromboprophylaxis. We supplemented our database searches by reviewing reference lists of key articles and examining clinical practice guidelines from major cardiovascular and orthopedic organizations.

**Figure 1:**
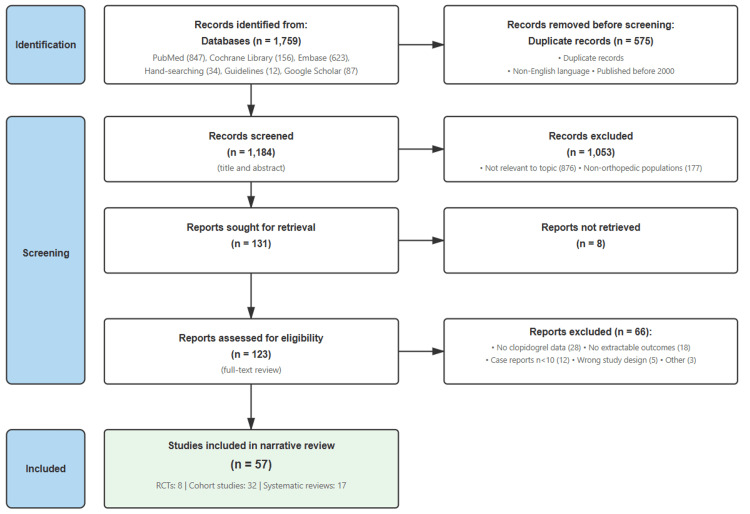
Study selection process showing the identification, screening, and inclusion of studies for this focused narrative review RCTs: randomized controlled trials

We focused our review on studies that examined adult orthopedic patients taking clopidogrel who required VTE prophylaxis, prioritizing research that reported clinically relevant outcomes such as bleeding complications, VTE events, transfusion requirements, and mortality. We included randomized controlled trials, prospective and retrospective cohort studies, and systematic reviews that provided data on these outcomes. We excluded case reports with fewer than 10 patients, studies that did not specifically address clopidogrel use, and publications not available in English. The study selection process is shown in Figure 1.

We organized this review to answer practical questions surgeons face daily. Why doesn't clopidogrel prevent blood clots in the veins? When should we stop it before surgery? Is it safe to use clopidogrel along with LMWH? How do we manage emergency surgeries when patients can't stop their clopidogrel? We included studies that looked at real patient outcomes, not just laboratory tests. The goal was to create practical recommendations that balance the risk of bleeding against the risk of dangerous blood clots. We prioritized evidence that directly applies to orthopedic patients rather than general medical patients.

We assessed the quality of included randomized trials using the Cochrane Risk of Bias tool and evaluated cohort studies using the Newcastle-Ottawa Scale. Given the substantial heterogeneity in study populations (elective arthroplasty vs. trauma patients), interventions (variable clopidogrel cessation protocols and LMWH dosing), and outcome definitions (different bleeding classification systems), we determined that formal meta-analysis would produce misleading pooled estimates. Where comparable studies existed, we calculated I² statistics and found values of 71-82%, confirming substantial statistical heterogeneity. We therefore conducted narrative synthesis, organizing findings by clinical question and reporting individual study statistics (p-values, confidence intervals, effect sizes) in evidence tables. This approach preserves the clinical context necessary for surgical decision-making while systematically presenting all available evidence.

Arterial versus venous thrombosis: why one drug cannot replace the other

Arterial and venous clots are fundamentally different (Table 1).

**Table 1:** Arterial vs. venous thrombosis DVT: deep vein thrombosis; PE: pulmonary embolism; LMWH: low-molecular-weight heparin; P2Y12: purinergic receptor subtype (platelet receptor that clopidogrel blocks); Xa: factor 10-a (coagulation factor)

Characteristics	Arterial thrombosis (white thrombi)	Venous thrombosis (red thrombi)
Flow conditions	High pressure, high flow	Low pressure, low flow
Primary mechanism	Endothelial injury → platelet adhesion	Stasis + hypercoagulability → coagulation cascade
Clot composition	Platelet-rich, fibrin-reinforced	Fibrin-rich, red blood cell entrapment
Key mediators	Collagen, von Willebrand factor, platelet aggregation	Coagulation factors (especially Xa, thrombin)
Clinical manifestations	Myocardial infarction, stroke, peripheral arterial occlusion	DVT, PE
Preventive medication	Clopidogrel (P2Y12 inhibitor)	LMWH (enhances antithrombin)
Drug mechanism	Irreversibly blocks platelet P2Y12 receptor (7-10-day effect)	Binds antithrombin → inactivates factor Xa and thrombin

They are formed through different mechanisms based on the type of blood flow within the vessel. Arterial thrombosis (white thrombi) occurs in high-pressure and high-flow systems. These clots develop due to injury to the endothelium (the layer that lies inside the vessels). This injury triggers platelet adhesion and activation. The exposed collagen and von Willebrand factor promote rapid platelet aggregation, producing a platelet-rich plug reinforced by fibrin. Arterial clots occlude arteries, causing myocardial infarction, stroke, and peripheral arterial occlusion [5,6].

Venous thrombosis (red thrombi) occurs in low-pressure and low-flow systems. Venous clots form when blood stasis and hypercoagulability activate the coagulation cascade. This leads to the generation of fibrin strands that entrap large numbers of red blood cells, creating a clot that is rich in fibrin and erythrocytes. They can cause DVT and pulmonary embolism (PE). This mechanism involves coagulation factor activation rather than platelet aggregation [7,8].

Clopidogrel impairs primary hemostasis by irreversibly blocking the P2Y12 receptor on platelets. This impairs platelet activation and aggregation. The effect lasts for the entire 7-10-day lifespan of platelets. This mechanism is highly effective against arterial thrombosis, which is mainly formed from platelets, but is ineffective against venous clots [9-13]. LMWH blocks secondary hemostasis. It binds to antithrombin, increasing its ability to inactivate factor Xa and thrombin. This interrupts the coagulation cascade and leads to venous thrombosis [14].

These complementary mechanisms increase the risk of bleeding when both drugs are combined, making it difficult to control bleeding. Despite the increased risk of bleeding, this combination is often necessary. PE carries a 10-30% mortality if untreated. DVT causes significant morbidity, including post-thrombotic syndrome. The bleeding risk from combination therapy is generally acceptable compared to these serious thrombotic complications. Individual risk assessment guides this decision [15].

Multiple studies and clinical guidelines have established that clopidogrel is ineffective in preventing VTE. Although it effectively reduces arterial events by inhibiting platelet aggregation, it does not adequately address the coagulation cascade. Evidence in orthopedic and hospitalized patients confirms that those on clopidogrel who become immobile remain at significant risk of DVT and PE, with incidence rates comparable to patients who receive no prophylaxis. Therefore, leading clinical recommendations consistently assert that clopidogrel should not be used in place of standard anticoagulant prophylaxis [16-19].

Studies specifically examining clopidogrel for VTE prevention in orthopedic patients are limited. The Pulmonary Embolism Prevention (PEP) trial was one of the largest studies examining antiplatelet therapy for VTE prevention [20]. Over 17,000 patients with hip fractures were enrolled. Even aspirin, which has some VTE prevention effects, only reduced PE by 43% and DVT by 29%. This partial protection is inadequate in high-risk orthopedic patients [21-23]. Clopidogrel has even less evidence for VTE prevention than aspirin. There are no major trials showing its effectiveness in preventing venous clots in immobilized patients. Without any prophylaxis, historical data show DVT rates of 40-60% after hip and knee replacement surgery, with some sources reporting up to 80% in orthopedic surgical patients. With LMWH prophylaxis, total VTE rates (symptomatic and asymptomatic) drop to approximately 0.6-1.5% [19,24,25]. Table 2 presents the key evidence demonstrating why clopidogrel cannot replace LMWH for VTE prophylaxis in orthopedic patients.

**Table 2:** Evidence that clopidogrel does not prevent VTE VTE: venous thromboembolism (includes DVT and PE); DVT: deep vein thrombosis; PE: pulmonary embolism; RCT: randomized controlled trial; LMWH: low-molecular-weight heparin

Study/source	Type	Population (n)	Key finding	VTE rates
PEP Trial, 2000 [20]	Large RCT	Hip fracture (17,444)	Aspirin reduced DVT by only 29% and PE by only 43% compared to placebo	Even aspirin provides inadequate VTE prophylaxis as monotherapy
Llau et al., 2018 [16]	European guidelines	Surgical patients	Antiplatelet agents should not replace anticoagulant prophylaxis for VTE prevention	Clinical recommendation based on the mechanism of action
Diep and Garcia, 2020 [17]	Systematic review	Various populations	Clopidogrel has no established benefit for VTE prevention; targets wrong mechanism	Antiplatelet agents ineffective for venous thrombosis
Spandonaro et al., 2013 [19]	Review	Orthopedic surgery	LMWH reduces VTE by 40-60% compared to no prophylaxis	With LMWH: 0.6-1.5% VTE rate

When to stop clopidogrel: clear indications

Certain procedures require stopping clopidogrel, when possible, five to seven days preoperatively. Major joint replacement, such as total hip replacement (THR), total knee replacement (TKR), revision joint arthroplasty, and spine surgery, always includes extensive tissue dissection and bone work, which is associated with a high risk of bleeding. Other considerations include procedures when tourniquet use is not applicable or contraindicated, and procedures with difficult surgical exposure have a higher risk of bleeding [26,27].

When stopping clopidogrel is contraindicated

The indication for clopidogrel determines whether its discontinuation is safe. The absolute contraindications for stopping clopidogrel are limited but critical. Patients with bare-metal stents require at least four weeks of therapy. Drug-eluting stents require 6-12 months, depending on the generation and clinical factors. Recent stent thrombosis while on therapy indicates a high risk. Recent acute coronary syndrome mandates 12 months of dual antiplatelet therapy. Stopping too early risks stent thrombosis with 40-50% mortality. These patients should not stop clopidogrel under any circumstances until they receive a cardiology evaluation before any surgical decision [28,29].

Managing clopidogrel in elective orthopedic surgery

For elective procedures, clopidogrel should be stopped five to seven days preoperatively. This allows the production of new functional platelets. By surgery day, approximately 70-80% of platelet function recovers. This level provides adequate hemostasis for most procedures while maintaining residual antiplatelet effects [30,31].

In situations where discontinuing clopidogrel is contraindicated, surgery should be delayed if possible. When a delay is unacceptable, multidisciplinary team (MDT) management is mandatory. Cardiologist input is crucial throughout the perioperative period. A full plan should be set for these patients with clear documentation of when to stop and restart the drug and if a drug substitution is necessary. Some patients may transition to aspirin alone if it is appropriate. Others may benefit from shorter-acting agents [32]. 

Managing clopidogrel in trauma surgery

Trauma patients cannot have planned medication cessation. Hip fractures are an example of such challenges. Delaying surgery beyond 36-48 hours significantly increases mortality. Delaying surgery for more than 48 hours leads to a 32% possibility of death within the first year [33,34]. Waiting five to seven days for platelet recovery is unrealistic and can be catastrophic. Therefore, surgery must be performed despite antiplatelet therapy.

Current evidence supports operating without delay. Multiple studies have shown acceptable outcomes in patients with hip fractures treated with clopidogrel. Transfusion requirements increase by 20-30%. Blood loss increases by 100-200 mL on average. However, serious bleeding complications remain rare. The survival benefit of early surgery far outweighs the bleeding risk [35-37].

The surgical team should be prepared for increased bleeding. Blood products should also be available. A meticulous surgical technique minimizes blood loss. Cell salvage may be useful in this scenario. Local or systemic tranexamic acid (TXA) injection reduces bleeding. From an anesthetic perspective, regional techniques such as spinal or epidural anesthesia are not advised if clopidogrel has not been stopped for the recommended period, as they carry a high risk of spinal hematoma. In these situations, general anesthesia is the safest choice [38-40].

After elective and trauma surgeries, the timing of restarting clopidogrel depends on the bleeding risk. Most patients resume clopidogrel 24-48 hours postoperatively. An earlier resumption may be appropriate for patients with a very high thrombotic risk. A delayed restart is reasonable if hemostasis concerns exist. In cases of uncertainty, a cardiologist's opinion should be sought to avoid patient harm [41].

Table 3 summarizes the key studies examining bleeding outcomes in both elective and trauma surgery settings, demonstrating the consistent pattern of increased blood loss and transfusion requirements with continued clopidogrel therapy.

**Table 3:** Key studies on clopidogrel and bleeding risk in orthopedic surgery THA: total hip arthroplasty (total hip replacement); TKA: total knee arthroplasty (total knee replacement); clop: clopidogrel; NS: not significant (p>0.05, no statistical significance); OR: odds ratio; HR: hazard ratio

Study	Design	Population (n)	Surgery type	Clopidogrel management	Blood loss (mL)	Transfusion rate	Major bleeding	Key findings
Wu et al., 2022 [26]	Retrospective cohort	186 (93 clop, 93 control)	THA/TKA	Continued perioperatively	Not reported	18.3% vs. 8.6% (p=0.02)	8.6% vs. 3.2% (p=0.04)	Increased bleeding and transfusion risk with continued clopidogrel
Nandi et al., 2012 [27]	Case-control	60 (30 clop, 30 control)	THA/TKA	Stopped 5-7 days pre-op	Not reported	23.3% vs. 13.3% (NS)	6.7% vs. 3.3% (NS)	Appropriate cessation period is safe with acceptable bleeding risk
Klestil et al., 2017 [33]	Systematic review	28 studies	Hip fracture	Variable timing	Meta-analysis: +150-200 mL	OR 1.4-1.6	3-9% range	Mortality increases significantly with surgical delay >48 hours (HR 1.32)
Zheng et al., 2021 [35]	Retrospective cohort	120 (60 clop, 60 control)	Hip fracture	Continued (surgery mean 2.8 days)	485±156 vs. 312±98 (p<0.001)	45% vs. 21.7% (p=0.008)	8.3% vs. 1.7% (p=0.08)	Early surgery safe despite increased bleeding; no mortality difference (3.3% vs. 5%)
Wallace et al., 2012 [36]	Case-control	38 (19 clop, 19 control)	Hip fracture	Continued (surgery mean 1.9 days)	375±198 vs. 280±145 (p=0.04)	52.6% vs. 31.6% (p=0.09)	10.5% vs. 5.3% (NS)	Acceptable bleeding risk; early surgery should not be delayed
Yang et al., 2021 [37]	Case-control	120 (60 clop, 60 control)	Hip fracture	Continued (surgery <48 hours)	441±145 vs. 298±102 (p<0.001)	41.7% vs. 20% (p=0.01)	5% vs. 1.7% (NS)	Early surgery feasible; mortality 1.7% vs. 3.3% (NS)

LMWH dosing with concurrent clopidogrel

Standard LMWH dosing applies to most patients taking clopidogrel. Enoxaparin 40 mg daily provides effective prophylaxis against VTE. Dalteparin 5000 units/day is an alternative. No routine dose reduction is recommended. Special populations may require adjusted dosing. Severe renal impairment requires dose reduction or the use of alternative agents. Patients with a history of severe bleeding may require extra caution. Individual assessments guide these modifications [42]. Extreme weight requires special consideration when combining these medications. Underweight patients (below 50 kg) have a higher bleeding risk due to relative overdosing; therefore, they may benefit from reduced doses. Obese patients (body mass index >40 kg/m²) may have inadequate prophylaxis with standard doses. They often require weight-based LMWH dosing. Anti-Xa monitoring can help optimize dosing in these challenging cases [43].

The timing of LMWH administration is critical in orthopedic patients, particularly those receiving antiplatelet therapy, such as clopidogrel. When LMWH is used preoperatively, the last prophylactic dose should be administered at least 12 hours before surgery to reduce the risk of bleeding. Administering LMWH closer to the time of surgery, especially in combination with clopidogrel, markedly increases the likelihood of bleeding. Therefore, many perioperative protocols favor initiating LMWH postoperatively, typically 12-24 hours after the procedure, once hemostasis is secured. In selected high-risk patients on dual antithrombotic therapy, initiation may be further delayed depending on the balance between bleeding and thrombotic risks [32]. If the bleeding risk is high, temporary use of mechanical methods such as intermittent pneumatic compression (IPC) or graduated compression stockings (GCS) is recommended until pharmacologic prophylaxis becomes safe, in line with the American College of Chest Physicians (CHEST) and National Institute for Health and Care Excellence (NICE) guidelines [44-47].

Practical considerations for combination therapy

Communication between specialties improves outcomes in complex patients. The orthopedic team needs to be aware of the cardiac indications for clopidogrel. Cardiologists must understand the risks of surgical bleeding. Anesthetists require information on medication timing to ensure safe neuraxial blockade. Pharmacists can identify drug-drug interactions and dosing issues. Clear documentation can prevent medication errors. This multidisciplinary team approach ensures that all aspects of care are coordinated.

Patient education is essential for the safe management of this condition. Patients must understand why both medications are necessary. They should be aware of the signs of bleeding and report them immediately. Instructions regarding medication timing must be clear. Written materials can help prevent confusion. Family members should be included in education, when possible. Patients must know who to contact with their concerns. Good communication prevents complications and improves compliance.

Clinical monitoring is more valuable than laboratory testing in patients receiving LMWH and antiplatelet therapy. Daily hemoglobin checks can detect occult bleeding. Surgical site assessment can identify hematomas early. The drain output requires close monitoring. Patient symptoms, such as dizziness or tachycardia, may indicate bleeding. This clinical vigilance allows for early intervention before major complications develop.

TXA in patients on antiplatelet therapy

TXA is an antifibrinolytic drug that helps stabilize blood clots and reduce surgical bleeding. It is widely used in orthopedic procedures to reduce blood loss and transfusion requirements. In orthopedic patients taking clopidogrel, the theoretical concern is that adding TXA might tip the hemostatic balance toward thrombosis, especially when LMWH prophylaxis is delayed or reduced due to bleeding risk. However, evidence from hip fracture cohorts shows that TXA consistently lowers blood loss and transfusion rates without increasing symptomatic VTE or mortality [48-50]. Guidelines for hip fractures and arthroplasty now endorse TXA as part of standard blood-sparing care [51]. Where antiplatelet therapy cannot be interrupted, a practical compromise is to prefer topical TXA (or limited-dose IV) to reduce systemic exposure; a small series suggests that topical TXA retains efficacy in patients continuing aspirin [52]. In patients on dual antiplatelet therapy, high-quality data remain unclear; individualized decisions should consider the bleeding phenotype, timing of LMWH (post-op start when hemostasis is secure), and use of mechanical prophylaxis until pharmacologic prophylaxis is safe. Table 4 summarizes the evidence on TXA safety and efficacy in patients receiving antiplatelet therapy, showing consistent blood loss reduction without increased VTE risk.

**Table 4:** TXA safety and efficacy in patients on antiplatelet therapy TXA: tranexamic acid; CI: confidence interval (usually 95% CI); RR: risk ratio (also called relative risk); OR: odds ratio; SMD: standardized mean difference; VTE: venous thromboembolism; NS: not significant (p>0.05); THA: total hip arthroplasty

Study	Design	Population (n)	TXA protocol	Blood loss reduction	Transfusion rate	VTE events	Key findings
Augustinus et al., 2022 [48]	Meta-analysis	2,847 (14 studies)	Topical and/or IV	Mean difference: -182 mL (95% CI: -268 to -96)	RR: 0.57 (95% CI: 0.47-0.70)	1.8% vs. 2.1% (NS)	TXA safe and effective; no VTE increase
Khan et al., 2025 [49]	Retrospective	200 (100 TXA, 100 control)	IV: 1 g pre-incision + 1 g at 3 hours	287±98 vs. 412±156 mL (p<0.001)	18% vs. 34% (p=0.008)	2% vs. 3% (NS)	Significant blood-sparing effect without thrombotic complications
Liu and Lee, 2025 [50]	Meta-analysis	3,156 (21 studies)	Various (topical/IV/oral)	SMD: -0.89 (95% CI: -1.24 to -0.54)	OR: 0.42 (95% CI: 0.32-0.54)	OR: 1.12 (95% CI: 0.68-1.85, NS)	TXA efficacious without increasing VTE or mortality
Huerfano et al., 2020 [52]	Cohort	134 revision THA	Topical: 3 g in 100 mL saline	432±298 vs. 687±445 mL (p<0.001)	24% vs. 43% (p=0.01)	0% vs. 1.5% (NS)	Topical TXA effective even in patients on aspirin (86% of cohort)

Focused clinical practice points for orthopedic surgeons

Based on the current evidence, several practical recommendations can guide orthopedic surgeons when managing patients on clopidogrel who also require LMWH.

Elective Surgery

Stop clopidogrel 5-7 days before the operation when safe, especially in patients without a very high thrombotic risk. A cardiologist should always be involved if stents or recent acute coronary syndromes are present.

Trauma Surgery (e.g., Hip Fractures)

Surgery should not be delayed beyond 36-48 hours because mortality increases with each day of delay. Surgery should be performed with preparation for increased bleeding, and blood products should be available.

Anesthesia

Spinal or epidural anesthesia should be avoided unless clopidogrel has been stopped for the recommended interval; general anesthesia is safer in urgent trauma cases.

LMWH Timing

The last prophylactic dose should be ≥12 hours before surgery; restart 12-24 hours postoperatively, once hemostasis is secured.

Mechanical Prophylaxis

Use IPC or GCS temporarily when the bleeding risk is too high for anticoagulation.

Restarting Clopidogrel

Clopidogrel is restarted usually within 24-48 hours postoperatively, earlier if thrombotic risk is very high, and later if bleeding risk remains significant.

Future directions in perioperative antithrombotic care

First, perioperative "bridging" for patients at a very high risk of ischemic events is evolving: short-acting IV cangrelor can maintain P2Y12 inhibition until a few hours pre-op, with 2024 perioperative guidance highlighting cardiology-led use in carefully selected cases [53]. Second, a specific reversal agent for ticagrelor (bentracimab) has shown positive phase 3 results, rapidly restoring platelet function in patients requiring urgent surgery or those with major bleeding. Regulatory availability will shape future pathways, although clopidogrel still lacks a targeted antidote [54]. Third, "precision prophylaxis" is emerging: machine learning models to predict post-arthroplasty VTE/bleeding risk could individualize agent choice and duration [10,11], while genotype-guided antiplatelet strategies (CYP2C19) may help select alternatives to clopidogrel for poor/intermediate metabolizers in the cardiac population feeding into orthopedic workflows [55,56]. Finally, dosing calculation continues to mature: for obesity and trauma, anti-Xa-guided or weight-based LMWH strategies may improve prophylactic target attainment, although routine anti-Xa monitoring has not consistently improved outcomes outside select settings [57].

## Conclusions

LMWH and clopidogrel target different thrombotic mechanisms. Clopidogrel prevents arterial clot formation but provides inadequate protection against venous thrombosis. Orthopedic patients taking clopidogrel still require LMWH for VTE prevention. In both elective and trauma cases, the indication and timing of clopidogrel administration determine whether it should be stopped. Decisions must balance the benefits of early surgery against the bleeding risks of dual therapy. Careful risk assessment, cardiology consultation, and close collaboration between surgeons and anesthetists guide the safest approach.
